# Attention-Based Fault-Tolerant Approach for Multi-Agent Reinforcement Learning Systems

**DOI:** 10.3390/e23091133

**Published:** 2021-08-31

**Authors:** Shanzhi Gu, Mingyang Geng, Long Lan

**Affiliations:** 1 College of Computer, National University of Defense Technology, Changsha 410073, China; gsznudt11@gmail.com (S.G.); gengmingyang13@nudt.edu.cn (M.G.); 2 High Performance Computing Laboratory, National University of Defense Technology, Changsha 410073, China

**Keywords:** reinforcement learning, attention mechanism, fault tolerance, multi-agent

## Abstract

The aim of multi-agent reinforcement learning systems is to provide interacting agents with the ability to collaboratively learn and adapt to the behavior of other agents. Typically, an agent receives its private observations providing a partial view of the true state of the environment. However, in realistic settings, the harsh environment might cause one or more agents to show arbitrarily faulty or malicious behavior, which may suffice to allow the current coordination mechanisms fail. In this paper, we study a practical scenario of multi-agent reinforcement learning systems considering the security issues in the presence of agents with arbitrarily faulty or malicious behavior. The previous state-of-the-art work that coped with extremely noisy environments was designed on the basis that the noise intensity in the environment was known in advance. However, when the noise intensity changes, the existing method has to adjust the configuration of the model to learn in new environments, which limits the practical applications. To overcome these difficulties, we present an Attention-based Fault-Tolerant (FT-Attn) model, which can select not only correct, but also relevant information for each agent at every time step in noisy environments. The multihead attention mechanism enables the agents to learn effective communication policies through experience concurrent with the action policies. Empirical results showed that FT-Attn beats previous state-of-the-art methods in some extremely noisy environments in both cooperative and competitive scenarios, much closer to the upper-bound performance. Furthermore, FT-Attn maintains a more general fault tolerance ability and does not rely on the prior knowledge about the noise intensity of the environment.

## 1. Introduction

Consider the following robotic search and rescue scenario: A group of Unmanned Aerial Vehicles (UAVs) is sent to find the survivors in a group of high-rise buildings after an earthquake [1]. The harsh environmental conditions might cause individual robots to fail, or hackers might take control of some robots and force them to behave in misleading ways [2]. In order to find the survivors as quickly as possible, these robots have to periodically exchange information with the neighbors and make decisions based both on the local view and the correct information from the neighbors.

**Figure 1:**
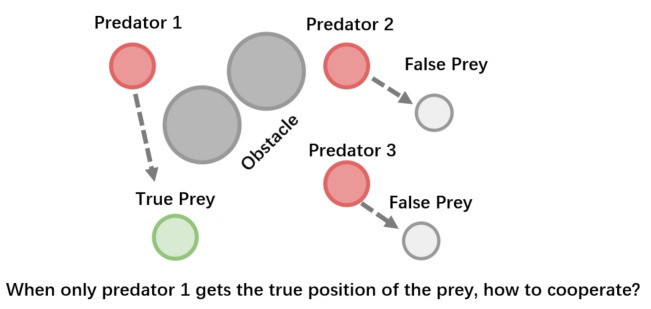
An illustration of our scenario: the modified predator and prey problem. Three slower predators learn to cooperate to capture a faster prey with obstacles impeding the way. However, when both Predator 2 and Predator 3 obtain the wrong relative position of the prey, the learning process will become extremely difficult since they must learn to trust Predator 1 along with learning the action policies. All the agents are not aware of whether they have faulted or not.

The above-mentioned multirobot cooperation scenario can be modeled as a Multi-Agent Reinforcement Learning (which we refer to as *MARL*) problem. MARL systems aim to provide interacting agents with the ability to collaboratively learn and adapt to other agents’ behaviors. Plenty of real-world applications can be modeled as multi-agent systems, e.g., autonomous driving [3], smart grid control [4], and multirobot control [5,6,7,8,9,10,11]. Typically, an agent receives its private observations providing a partial view of the true state of the world. However, in realistic settings, one or more agents that show arbitrarily faulty or malicious behavior may suffice to allow the current coordination mechanisms fail [12]. An illustrative example is shown in Figure 1: three slower predators (red circles) learn to cooperate to capture a faster prey (the green circle) with obstacles (dark blue circles) impeding the way. However, when Predator 2 and Predator 3 obtain the wrong relative position of the prey (light grey circles), the learning process will become extremely difficult. In concrete, the predators must reach an agreement on whom to trust along with learning the action policies. Therefore, fault tolerance and credit assignment have become of paramount importance in noisy MARL systems.

The existing work on the Multi-agent Deep Deterministic Policy Gradient-Medium (MADDPG-M) [13] has achieved state-of-the-art performance in extremely noisy environments. However, the MADDPG-M is designed on the basis that the noise intensity in the environment is known in advance. When the noise intensity changes, the MADDPG-M has to adjust the configuration of the broadcasting medium to learn in the new environment, which limits its practical applications. Furthermore, the MADDPG-M can only select the correct information in the extremely noisy environments and cannot make a finer discrimination (i.e., judge whether the correct information is relevant to a specified agent or not). Therefore, enabling the agents to collaboratively solve the cooperation tasks with a more general fault tolerance ability is necessary, and we consider two challenges to achieve this. First, a proper information filtering mechanism needs to be designed for the agents to extract not only correct, but also relevant information from others and model the environment without the restriction of noise intensity. Then, the information filtering mechanism should maintain a stable complexity while keeping the ability to deal with different kinds of uncertainties in the environment, i.e., accommodate various numbers of agents with noisy observations without tuning the configuration of the model. In concrete, the model should maintain the ability to deal with the complex cases where an agent needs to reach multiple agents’ correct observations at the same time without the prior knowledge of the noise intensity of the environments.

To overcome the challenges, we present an Attention-based Fault-Tolerant (FT-Attn) model, which can select not only correct, but also relevant information for each agent at every time step. The multihead attention mechanism enables the agents to learn effective communication policies through experience concurrently with the action policies. Rather than simply sharing the specified number of correct observations, FT-Attn estimates the critic function for a single agent by selecting and utilizing the useful encoded information from others without the restriction on the filter mechanism. We study the performance of FT-Attn in the modified cooperative navigation and modified predator and prey environments and compare our results with the previous state-of-the-art method, the MADDPG-M [13]. The results show a clear advantage of FT-Attn in some extremely noisy environments of both cooperative and competitive scenarios compared with the baselines. Furthermore, FT-Attn maintains a more general fault tolerance ability and does not rely on the prior knowledge about the noise intensity of the environment. In other words, FT-Attn can be directly utilized to learn in various kinds of noisy environments with no need to tune the configuration of the model, while the MADDPG-M has to adjust the amount of information to be shared among agents. We also visualize the attention weights generated by FT-Attn to inspect how the fault tolerance mechanism is working. To the best of our knowledge, we are the first to apply the attention mechanism to cope with the fault tolerance problem in MARL systems. We mention that FT-Attn is not designed for competing with other models without considering fault tolerance, but a complementary one. We believe that adding our idea of fault tolerance will make the existing models much more valuable and practical.

The rest of this paper is organized as follows. Section 2 introduces the related work of MARL methods in normal and noisy environments. Section 3 introduces the background of partially observable Markov games and basic reinforcement learning algorithms. Section 4 describes the methodology of our work, as well as the architecture designed for training and prediction. The validation and evaluation of our work in the modified cooperative navigation and the modified predator and prey environments is described in Section 5. The limitations of our approach are discussed in Section 6. We conclude and provide our future directions in Section 7.

## 2. Related Work

MARL has been studied for a long time [14]. There are diverse kinds of topics within Multi-agent Reinforcement Learning (MARL), including learning communication between cooperative agents [15] and algorithms for optimal play in competitive settings [16]. The MARL models can be divided into two kinds: designed for normal environments and designed for noisy environments.

### 2.1. MARL Models Designed for Normal Environments

In the early stage, References [17,18] studied MARL with decentralized execution. However, these kinds of methods cannot be applied to complex environments since they are based on tabular methods. With the development of deep learning technologies, plenty of deep MARL algorithms [19,20,21] have been proposed. We now introduce several competing models in chronological order. The Communication Neural Network (CommNet) [22] is devoted to obtaining an integrated communication vector for each agent by average pooling over all messages sent from the other agents. The drawback is that the CommNet does not perform a fine discrimination of the messages passed by the agents. Differentiable Inter-Agent Learning (DIAL) [23] aims to solve simple communication tasks such as guessing riddles, but it cannot solve the problem in nonstationary environments. The Bidirectional-coordinated Net (BicNet) [24] was proposed to handle real-time strategy games such as StarCraft. A drawback is that it assumes the agents are fully observable for the environment, which limits its practice in reality. The Multi-agent Deep Deterministic Policy Gradient (MADDPG) [25] first extended the traditional actor–critic algorithms to the multi-agent coordination setting. However, it solves coordination by directly introducing the observations and actions of other agents without filtering, which may lead to the problem of excessive state space. The Attentional Communication Model (ATOC) [26] focuses on learning an attention model for sharing information between the policies and can be applied to large-scale systems with hundreds of agents. To incorporate a filter mechanism on the basis of the MADDPG, the Multi-Actor-Attention-Critic (MAAC) [27] utilizes an actor–critic algorithm that trains decentralized policies, using centrally computed critics that share an attention mechanism. The Scheduling Network (SchedNet) [28] aims to solve the MARL problem in limited-bandwidth environments. In concrete, SchedNet produces a weight *w* for each agent, and the top *k* agents in terms of their weights *w* can share their observations with the others. However, it is not practical to apply a hard-coded parameter *k* to real-world multi-agent settings since the bandwidth usually cannot be known in advance.

### 2.2. MARL Models Designed for Noisy Environments

For noisy environments, the Multi-agent Fault-tolerant Reinforcement Learning algorithm (MAFTRL) [29] establishes the agent’s own error detection mechanism and designs the information communication medium between agents. Besides, the MADDPG-M [13] addresses the MARL problem characterized by partial and extremely noisy observations, i.e., only one agent’s observation is correct. To deal with the noisy observations that are weakly correlated with the true state of the environment, the MADDPG-M forces the agents to learn whose private observation is sufficiently informative to be shared with others. However, the communication policy is task-specific, relying on prior knowledge about the noise intensity, which simplifies the uncertainties of the environments. Furthermore, the complexity of the MADDPG-M is compounded by a specific experimental evaluation. In concrete, if the experimental setting changes, MADDPG-M must adjust the configuration of the information filtering mechanism to learn in the new environments. Besides, when there exist multiple correct observations (in more general fault-tolerant scenarios), the MADDPG-M cannot select the relevant information for each agent on the basis of correct observations. In other words, the MADDPG-M cannot fully exploit the useful information provided by the environment and may lead to suboptimal performance. Additionally, the observation-sharing mechanism in practice may introduce redundant information (e.g., pixel data) because the raw observations may be high-dimensional. In our work, FT-Attn utilizes the attention mechanism to enable the MARL system to be fault-tolerant without the prior knowledge about the noise intensity in the environment.

## 3. Background

We start by introducing the basic building blocks for our approach: partially observable Markov games, policy gradient and actor–critic algorithms.

### 3.1. Partially Observable Markov Games

We consider the framework of Partially Observable Markov Games (POMGs) [16], which is a multi-agent extension of Markov decision processes. The framework contains *N* agents with partial observations and is characterized by a collection of true states *S*, a set of actions $A\;=\;\left\{A_{1},\cdots ,A_{N}\right\}$, a reward function *R*, a state transition function τ, a set of private observation functions $Q\;=\;\left\{Q_{1},\cdots ,Q_{N}\right\}$, a set of private observations $O\;=\;\left\{O_{1},\cdots ,O_{N}\right\}$, and a discount factor γ∈[0,1). Then, a POMG can be defined by a tuple, G = <S,A,τ,R,Q,O,γ,N>. Each agent can only receive a partial observation correlated with the true state and cannot gain full access to the whole true state of the environment s∈S, i.e., $o_{i}\;=\;Q_{i}\left(s\right):S\to O_{i}$. Each agent can take an action based on its own partial observation according to its action policy $\theta_{i}$, i.e., $a_{i}\;=\;\mu_{\theta_{i}}:O_{i}\to A_{i}$, and receives a reward, i.e., $r_{i}\;=\;R\left(s,a_{i}\right):S\times A_{i}\to R$. Then, the environment will move into the next state $S^{\prime }$ according to the state transition function conditioned on the actions of all agents, i.e., $s^{\prime }\;=\;\tau \left(s,a_{1},\cdots ,a_{N}\right):S\times A_{1}\times \cdots \times A_{N}\to S$. The aim of each agent is to maximize its own total expected return, $E\left[R_{i}\right]\;=\;E\left[\sum_{t\;=\;0}^{T}\gamma^{t}r_{i}^{t}\right]$, where *T* denotes the specified time step and $r_{i}^{t}$ denotes the reward received by the *i*-th agent at time *t*.

### 3.2. Policy Gradient and Actor–Critic

The aim of the policy gradient algorithm is to estimate the gradient of an agent’s expected returns according to the parameters of its policy. The gradient estimate mechanism works as follows:

(1) $$\nabla_{\theta }J\left(\pi_{\theta }\right)\;=\;E_{a∼\pi_{\theta }}\left[\nabla_{\theta }\log\left(\pi_{\theta }\left(a_{t}|s_{t}\right)\right)\sum_{t^{\prime }\;=\;t}^{\infty }\gamma^{t^{\prime }-t}r_{t^{\prime }}\left(s_{t^{\prime }},a_{t^{\prime }}\right)\right].$$

Due to the reason that the term $\sum_{t^{\prime }\;=\;t}^{\infty }\gamma^{t^{\prime }-t}r_{t^{\prime }}\left(s_{t^{\prime }},a_{t^{\prime }}\right)$ in the policy gradient estimator usually leads to high variance, the actor–critic algorithm [30] is then proposed to solve the problem. The aim of the actor–critic algorithm is to alleviate the issue by replacing the estimator with a function approximation of the expected returns. In concrete, given a state and an action, one specific instance of actor–critic methods learns a function to estimate expected discounted returns, $Q_{\psi }\left(s_{t},a_{t}\right)\;=\;E\left[\sum_{t^{\prime }\;=\;t}^{\infty }\gamma^{t^{\prime }-t}r_{t^{\prime }}\left(s_{t^{\prime }},a_{t^{\prime }}\right)\right]$, and then learns through the temporal difference by minimizing the regression loss:

(2) $$\begin{array}{c} L_{Q}\left(\psi \right)\;=\;E_{s,a,r,s^{\prime }}\left[{\left(Q_{\psi }\left(s,a\right)-y\right)}^{2}\right],\\ \mathrm{where}\;y\;=\;r\left(s,a\right)+\gamma E_{a^{\prime }∼\pi \left(s^{\prime }\right)}\left[Q_{\bar{\psi }}\left(s^{\prime },a^{\prime }\right)\right], \end{array}$$

where $Q_{\bar{\psi }}$ denotes the target Q-value function.

## 4. Our Approach

In this section, we first introduce the problem formulation of our MARL fault-tolerant setting. Then, we illustrate the core part of our proposed method FT-Attn: the attention-based fault-tolerant mechanism. Finally, the detailed steps of the training procedure are described.

### 4.1. Problem Formulation

We considered partially observable Markov games and made the assumption that the observations received by some of the agents are noisy and weakly correlated with the true state, which makes learning optimal policies unfeasible. Denote the policy for agent *i* on all *N* private observations as $a_{i}\;=\;\mu_{i}\left(o_{1},\cdots ,o_{N}\right)$. The learning process of the individual policy $a_{i}$ is hard to complete because a large number of $o_{i}$ are uncorrelated with the corresponding true state *s*, i.e., the background information provides a poor representation of the current true state for the *i*-th agent. In concrete, the noises satisfying a certain distribution are applied to the observation (a vector of real number) of the agents. In order to solve this challenge, each agent has to explicitly and selectively exploit the correct and useful observations shared by other agents. In other words, the agents have to form a common cognition internally before they learn the action policy to cooperate. Due to the reason that the agents cannot discriminate between relevant and noisy information on their own, the ability to decide whom to trust must also be acquired through experience.

### 4.2. Attention-Based Fault-Tolerant Mechanism

**Figure 2:**
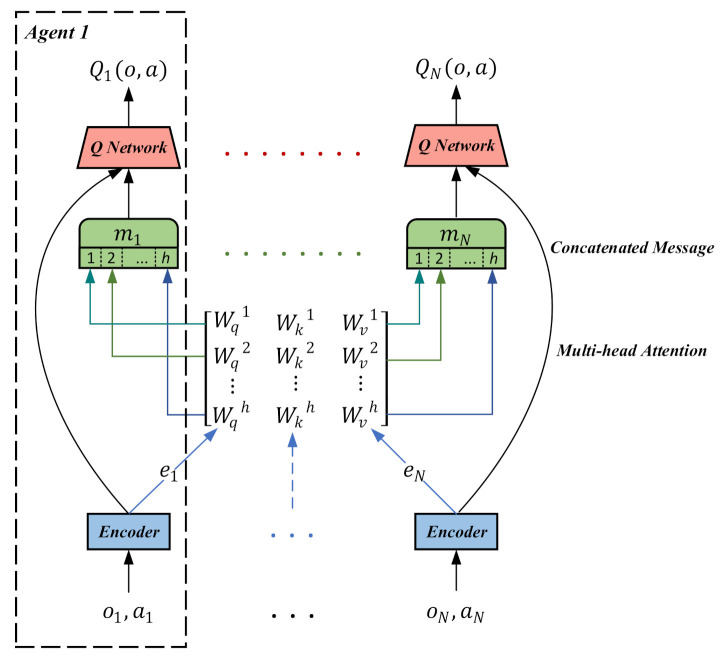
FT-Attn is composed of three modules: encoder, multihead attention-based information filtering part for fault tolerance, and Q-network.

More formally, we introduce our multi-head attention-based fault-tolerant mechanism to learn the critic for each agent by selectively paying attention to other agents’ observations. Figure 2 illustrates the main components of our approach. The multihead attention-based information filtering part is the core component of our approach to realize fault tolerance. We now describe the core part in detail.

We used multihead dot-product attention to select the correct and relevant observations for the agents in each time step. Intuitively, each agent inquires about other agents for information about their observations, as well as the actions and then takes the correct and relevant information into account for estimating its value function. Denote $Q_{i}^{\psi }\left(o,a\right)$ as the function of agent *i*’s observation and action, as well as other agents’ contributions; the value is estimated as follows:

(3) $$Q_{i}^{\psi }\left(o,a\right)\;=\;f_{i}\left(g_{i}\left(o_{i},a_{i}\right),m_{i}\right).$$

Here, $f_{i}$ represents the Q-network and $g_{i}$ represents the encoder function. Towards agent *i*, the correct and relevant message from other agents $m_{i}$ is a weighted sum of each agent’s value:

(4) $$m_{i}\;=\;\sigma \left(\;\mathrm{Concat}\;\left[\sum_{j\in \backslash i}\alpha_{ij}^{h}W_{v}^{h}e_{j},\forall h\in H\right]\right),$$

where *h* denotes an attention head and $e_{j}$ denotes the embedding encoded by the $g_{j}$ function. $W_{v}^{h}$ transforms $e_{j}$ into a “value”. The set of all agents except *i* is represented as \i and indexed with *j*. To calculate the interaction weight of the *h*-th attention head $a_{ij}^{h}$ between agent *i* and agent *j*, the input feature of each agent is projected to query, key, and value representation by each independent attention head. For attention head *h*, the relation between *i* and *j* is computed as:

(5) $$\alpha_{ij}^{h}\;=\;\frac{\exp\left(\tau \cdot W_{q}^{h}e_{i}\cdot {\left(W_{k}^{h}e_{j}\right)}^{⊤}\right)}{\sum_{r\in \backslash i}\exp\left(\tau \cdot W_{q}^{h}e_{i}\cdot {\left(W_{k}^{h}e_{r}\right)}^{⊤}\right)},$$

where τ is a scaling factor, $W_{q}^{h}$ transforms $e_{i}$ into a “query”, and $W_{k}^{h}$ transforms $e_{j}$ into a “key”. In the experiments, the multihead attention mechanism was exploited to generate an aggregated contribution, which contains the correct and relevant information from all other agents to agent *i*. The idea behind the multihead mechanism is to make each head focus on a different weighted mixture of agents. Then, the contributions from all attention heads are concentrated as a single vector to represent the correct and relevant message.

### 4.3. Training Details of FT-Attn

All critics are updated together to minimize a joint regression loss function because of the parameter sharing:

(6) $$L_{Q}\left(\psi \right)\;=\;\sum_{i\;=\;1}^{n}E_{(o,a,r,o^{\prime })∼D}\left[{\left(Q_{i}^{\psi }\left(o,a\right)-y_{i}\right)}^{2}\right],$$

where:

(7) $$y_{i}\;=\;r_{i}+\gamma E_{\alpha^{\prime }∼\pi_{\bar{\theta }}\left(o^{\prime }\right)}\left[Q_{i}^{\bar{\psi }}\left(o^{\prime },a^{\prime }\right)\right],$$

where $\bar{\psi }$ and $\bar{\theta }$ are the parameters of the target critics and target policies, respectively. $Q_{i}^{\psi }$, the action-value estimate for agent *i*, receives observations and actions for all agents. $Q_{i}^{\bar{\psi }}$ denotes the target Q-value function, which is simply an exponential moving average of the past Q-functions. *D* represents a replay buffer that stores past experiences. The individual policies are updated with the following gradient:

(8) $${▽}_{\theta_{i}}J\left(\pi_{\theta }\right)\;=\;E_{\alpha ∼\pi_{\theta }}\left[{▽}_{\theta_{i}}log\left(\pi_{\theta_{i}}\left(a_{i}|o_{i}\right)\right)\left(b\left(o,a_{\backslash i}\right)-Q_{i}^{\psi }\left(o,a\right)\right)\right],$$

where $b(o,a_{\backslash i})$ represents the multi-agent baseline used to calculate the advantage function. We sample all actions, *a*, from all agents’ current policies to calculate the gradient estimate for agent *i*. The multi-agent baseline is calculated by the following manner:

(9) $$\begin{array}{cc} b(o,a_{\backslash i}) & \;=\;E_{a_{i}∼\pi_{i}\left(o_{i}\right)}\left[Q_{i}^{\psi }\left(o,\left(a_{i},a_{\backslash i}\right)\right)\right]\\ & \;=\;\sum_{a_{i}^{\prime }\in A_{i}}\pi \left(a_{i}^{{}^{\prime }}|o_{i}\right)Q_{i}\left(o,\left(a_{i}^{\prime },a_{\backslash i}\right)\right), \end{array}$$

the advantage function here can help solve the multi-agent credit assignment problem [31]. In concrete, by comparing the value of specific action to the value of the average action for the agent, with all other agents fixed, we can know whether any increase in reward is attributed to other agents’ actions.

For completeness, the pseudocode for FT-Attn is provided in Algorithm 1. For the training procedure, our model was trained for $10^{5}$ episodes with 25 steps each episode. We added a tuple of ${\left(o_{t},a_{t},r_{t},o_{t+1}\right)}_{1\cdots N}$ to the replay buffer with a size of $10^{6}$ at each time step. We updated the network parameters after every 1024 tuples added to the replay buffer and performed gradient descent on the loss function. We used the Adam [32] optimizer with a learning rate of 0.001. For the other hyperparameters, the discount factor γ was set to 0.99; the dimension of the hidden state was set to 128; the number of attention heads was set to 4. For the exploration noise, following [33], we used an Ornstein–Uhlenbeck process [34] with θ = 0.15 and σ = 0.2.
**Algorithm 1** Training procedure for FT-Attn.**Input:** Initialize the environments with *N* agents; initialize replay buffer, *D*.1: **for**
$i_{ep}\;=\;1\cdots$ num episodes **do**2:   Observe initial state $o_{i}$ for each agent *i*,3:   **for** t = 1⋯ steps per episode **do**4:     Select actions $a_{i}∼\pi_{i}\left(\cdot |o_{i}\right)$ for each agent *i*.5:     Execute the action $a_{i}$ and get $o_{i}^{{}^{\prime }}$, $r_{i}$ for all agents.6:     Store transitions $\left(o_{1\cdots N},a_{1\cdots N},r_{1\cdots N},o_{1\cdots N}^{{}^{\prime }}\right)$ in *D*.7:     Sample minibatch $B\leftarrow m\times (o_{1\cdots N},a_{1\cdots N},r_{1\cdots N},o_{1\cdots N}^{{}^{\prime }})∼D$, and unpack.8:     Calculate $Q_{i}^{\psi }\left(o_{1\cdots N},a_{1\cdots N}\right)$ for all *i* in parallel, $a_{i}^{{}^{\prime }}∼\pi_{i}^{\theta }\left(o_{i}^{{}^{\prime }}\right)$, using target policies, $Q_{i}^{\bar{\psi }}\left(o_{1\cdots N}^{{}^{\prime }},a_{1\cdots N}^{{}^{\prime }}\right)$9:     Set $y_{i}\;=\;r_{i}+\gamma E_{\alpha^{{}^{\prime }}∼\pi_{\bar{\theta }}\left(o^{{}^{\prime }}\right)}\left[Q_{i}^{\bar{\psi }}\left(o^{{}^{\prime }},a^{{}^{\prime }}\right)\right]$,10:      Update critic by minimizing the loss:
(10)$$L_{Q}\left(\psi \right)\;=\;\sum_{i\;=\;1}^{n}E_{(o,a,r,o^{{}^{\prime }})∼D}\left[{\left(Q_{i}^{\psi }\left(o,a\right)-y_{i}\right)}^{2}\right]$$11:    Update policy:
(11)${▽}_{\theta_{i}}J\left(\pi_{\theta }\right)\;=\;E_{\alpha ∼\pi_{\theta }}\left[{▽}_{\theta_{i}}log\left(\pi_{\theta_{i}}\left(a_{i}|o_{i}\right)\right)\left(b\left(o,a_{\backslash i}\right)-Q_{i}^{\psi }\left(o,a\right)\right)\right]$12:    Update target critic and policy parameters:
(12)$$\bar{\psi }\;=\;\tau \bar{\psi }+\left(1-\tau \right)\psi$$
(13)$\bar{\theta }\;=\;\tau \bar{\theta }+\left(1-\tau \right)\theta$13:    **end for**14: **end for**

## 5. Experiments

To evaluate FT-Attn, we first introduce the experimental setting and baseline methods. Then, we show the experimental results compared with the baseline methods. Finally, we give the attention visualization and the corresponding analysis of FT-Attn.

### 5.1. Experimental Setting and Baseline Methods

To evaluate our proposed approach, we first chose two experimental settings: modified cooperative navigation and modified predator and prey [13] to test our approach in cooperative and competitive scenarios, respectively. The reason for selecting these two modified versions is that the original settings cannot satisfy the basic assumption of noisy environments. Therefore, we followed the experimental setting of [13] by adding noises to the observation of each agent for evaluation.

**Figure 3:**
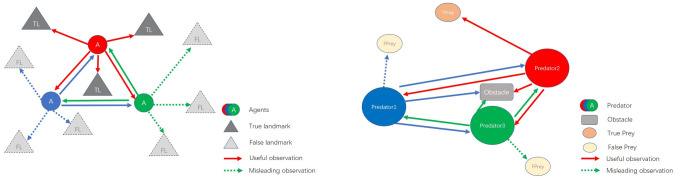
(**Left**) an illustration of the modified cooperative navigation problem: the gifted agent (red circle) can correctly observe all three landmarks (grey squares); the other agents (blue and green circles) receive the wrong locations of landmarks. (**Right**) an illustration of the modified predator and prey problem: the gifted predator (red circle) can correctly observe the position of the prey, while the other two predators receive the wrong location of the prey.

In the original cooperative navigation environment [25], *N* agents have to cooperate through actions to reach *L* landmarks. The agents can observe the relative positions of the other agents and landmarks. The agents are collectively rewarded according to the shortest proximity of any agent to each landmark. The agents must learn to infer the landmark they need to cover cooperatively and move there without colliding with other agents. For the modified version, as shown in the left part of Figure 3, we followed the setting in MADDPG-M [13]. In concrete, only 1 gifted agent of *N* agents can observe the true position of the landmarks, and all the other agents receive inaccurate information about the landmarks’ positions. In other words, there exist noises in the other 2 faulted agents’ observations on the relative positions of the landmarks. The task includes three different variants of increasing complexity depending on how the gifted agent is defined: in the “fixed” case, the gifted agent stays the same throughout the training phase; in the “alternating” case, the gifted agent may change at the beginning of each episode; in the “dynamic” case, which is the most difficult version, the agent closest to the center of the map becomes the gifted one within each episode. In other words, the agents must learn the underlying rule and reach an agreement on whom to trust before mastering the action policies.

In the original predator and prey task [25], there are *N* slower predators who must collaborate to capture a randomly faster prey with *L* obstacles impeding the way. Agents’ observations include the relative positions and velocities of all the other agents. Each time the cooperative predators collide with a prey, the predators are rewarded while the prey is penalized. We employed 3 predators and 1 prey in our experiment. In our modified version, as shown in the right part of Figure 3, only 1 gifted predator can obtain the correct relative position of the prey. In other words, there exist noises in the other 2 faulted agents’ observations on the relative positions of the prey. Therefore, the predators must learn to trust the gifted predator and infer the true position of the prey through the relative positions. The modified task also includes three different variants of increasing complexity: fixed, alternating, and dynamic versions.

For the baseline methods, we evaluated FT-Attn against five actor–critic-based baselines, the Deep Deterministic Policy Gradient (DDPG) [33], the Multi-agent Deep Deterministic Policy Gradient (MADDPG) [25], the meta-agent [13], the DDPG with Optimal Communication (DDPG-OC) [13], and the MADDPG-M [13]. The agents in the DDPG were trained and executed in a decentralized manner. The agents in the MADDPG were also executed in decentralized manner, but were trained in a centralized manner. The DDPG and MADDPG were chosen to provide a lower bound of the performance since each agent’s policy was conditioned only on its own observations. The meta-agent method was included to test the performance where all the observations were shared without a filtering mechanism. In concrete, a meta-agent can gain access to all the observations, across all agents both in the training and execution process. The DDPG-OC baseline utilizes a hard-coded communication pattern, i.e., the attention weights are assigned optimally using prior knowledge about the true environment. As the correct communication mechanism is identified in advance, the agents only need to learn the action policies in this case. The DDPG-OC was chosen to study what level of performance is achievable when communicating optimally in environments with different noise intensities. The MADDPG-M enables concurrent learning of the optimal communication policy and the underlying task, which enforces the agents to simply trust the agent with the largest sharing desire.

### 5.2. Performance Comparison in a Modified Cooperative Navigation Scenario

**Figure 4:**
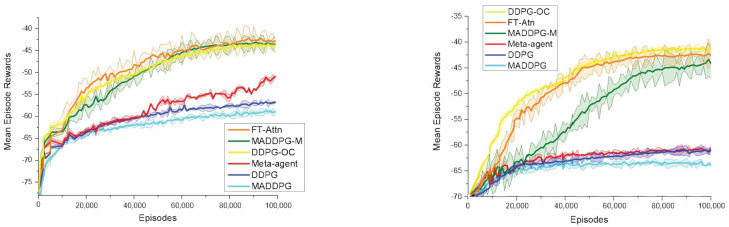
(**Left**) learning curves for all models in the alternating version of the modified cooperative navigation scenario. (**Right**) learning curves for all models in the dynamic version of the modified cooperative navigation scenario.

We first followed the experimental settings in [13], which set N = 3 and L = 3. Figure 4 illustrates the learning curves for FT-Attn and the other baseline methods on the alternating and dynamic scenario in terms of the mean episode rewards. In these cases, neither the DDPG nor the MADDPG can master the correct communication and action policies because they do not allow the observations to be shared. Furthermore, impacted by the meaningless actions of the faulted agents, even the gifted agent cannot master a rational action policy. In the test process, we found that the agents of these two methods only simply move towards the middle of the environment and then wander. The performance of the DDPG-OC illustrated an upper bound of each experimental setting since the correct communication policy was specified in advance. For the meta-agent method, the performance decreased sharply as the complexity of the environment increased and totally failed to learn in the dynamic setting. The result demonstrated that a filtering mechanism is necessary and the agents need to select the correct information to master the action policies. Conversely, both the MADDPG-M and FT-Attn can learn a rational behavior because of the message-selecting mechanism. However, the performance of FT-Attn was better than that of the MADDPG-M in both the alternating and dynamic settings, much closer to the upper-bound performance. The results showed that FT-Attn enables the agents to select more correct and relevant information to learn more effective communication policies in extremely noisy environments.

We ran an additional 1000 episodes after training to collect the performance metrics and report the averages for the three scenarios. Table 1 shows the mean episode rewards for FT-Attn and all baselines in the three scenarios. FT-Attn beat the MADDPG-M in the complex cases (alternating and dynamic versions) and performed quite similarly to the the upper-bound DDPG-OC. The standard deviation within one method was large because of the difficulty in the experimental setting. Besides, the mean reward improvements could reflect the performance improvements sufficiently because the test experiments were implemented 1000 times. This superiority demonstrated that the multihead attention mechanism maintains a better ability to select correct and relevant information to deal with extremely noisy observations. In order to further investigate the superiority of FT-Attn compared with the MADDPG-M, we further tested the communication accuracies of the two methods in the three variants in Table 2. In concrete, we implemented an additional 1000 episodes and recorded the times when the true gifted agent was trusted by the other faulted agents. In FT-Attn, we judged by selecting the agent with the maximum attention weight. In the MADDPG-M, we just selected the agent with the maximum sharing desire. From Table 2, we can see that the communication accuracies of FT-Attn were higher than those of the MADDPG-M, especially in the dynamic setting.

In order to further test the fault tolerance ability of FT-Attn and the baseline methods, we set N = 5 and L = 5 and gradually decreased the number of faulted agents from 4 to 1 in the fixed setting. We ran an additional 1000 episodes after training to collect the mean episode rewards and report the averages on the four scenarios when there are different numbers of faulted agents. Table 3 shows the mean episode rewards for FT-Attn and all baselines on the four scenarios. From Table 3, we can see that FT-Attn can easily achieve a better performance compared with the MADDPG-M without tuning the configurations (i.e., with no need to tune the hyperparameters for sharing the top (*k*) observations in terms of the weight values). Furthermore, the superiority of FT-Attn against the MADDPG-M was more evident when the number of faulted agents decreases and much closer to the performance of the upper-bound DDPG-OC. The reason is that FT-Attn can further select the relevant information on the basis of the correct information, while the MADDPG-M can only learn to select the correct observations. Note that the performance improvements should be measured on the basis of the upper-bound performance (DDPG-OC). In other words, the performance of FT-Attn was much closer to the upper-bound performance compared with the MADDPG-M, although the improvements seemed not to be very large. The results demonstrated that FT-Attn can better exploit the correct and useful information provided by the environment and achieve the scores closest to the upper-bound DDPG-OC. Furthermore, the stable performance of FT-Attn demonstrated that FT-Attn can directly be used to learn cooperative behaviors in various kinds of noisy environments without tuning the configuration of the model.

### 5.3. Performance Comparison in the Modified Predator and Prey Scenario

In the modified predator and prey scenario, the predators learn to collaborate to surround and seize the prey, while the prey aims to perform temptation and evasion. In our experiments, we focused on the cooperation between the predators rather than the competition between the predators and prey. In other words, the predators need to reach an agreement on whom is the gifted predator and infer the true position of the prey by trusting the gifted agent. For each method, the predators and prey were trained together so the termination state was that the rewards of the two reached a balance.

**Figure 5:**
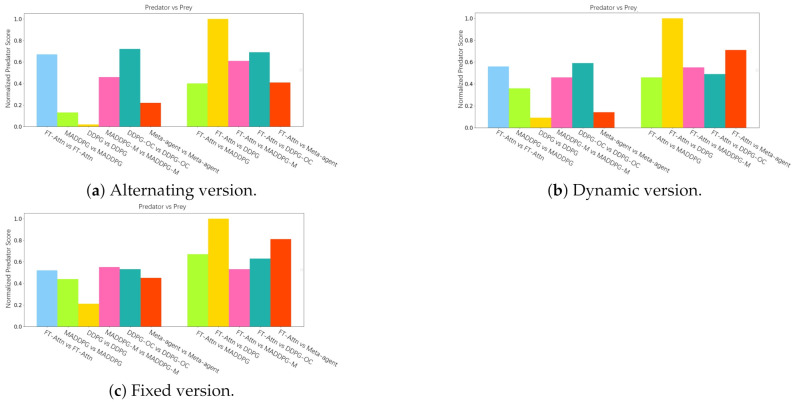
Cross-comparison between FT-Attn and the baseline methods in terms of the predator score in the different versions of the modified predator and prey scenario.

We implemented a cross-comparison between FT-Attn and the baseline methods to evaluate the policies learned. In concrete, we performed the modified scenario using predator and prey policies of the same method or FT-Attn predators against the baseline preys. As illustrated in Figure 5, we show the results in terms of the 0–1 normalized mean predator score of 30 test runs for the alternating, dynamic, and fixed version, respectively. For each subfigure, there exist two bar clusters divided from the middle. The first bar cluster illustrates the scenarios between the predators and prey of the same training method. We can see that although the environment was noisy, the scenario setting was always more suitable for predators since all the methods obtained positive predator scores. The second bar cluster illustrates the scores in the settings where the FT-Attn predators were against the MADDPG, DDPG, MADDPG-M, DDPG-OC, and meta-agent preys. The predator policy learned from FT-Attn beat that of the MADDPG-M in both the alternating and dynamic scenarios since the normalized score of FT-Attn vs. the MADDPG-M was higher than that of the MADDPG-M vs. the MADDPG-M. We can conclude that FT-Attn predators learn stronger and more robust policies than the baseline methods except the DDPG-OC, since FT-Attn predators obtained higher scores than the baselines when confronted with the same prey policy. Therefore, we argue that FT-Attn maintains a better fault-tolerant performance even in the competitive scenarios, and the learned policies in noisy environments can be generalized to the opponents with different policies.

### 5.4. Attention Visualization

**Figure 6:**
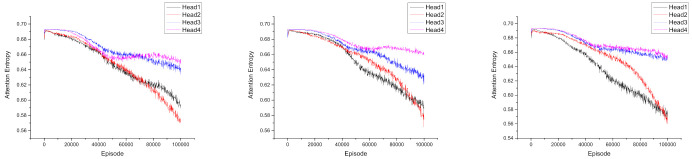
Attention entropy for each head over the course of training for the three agents in the “dynamic” situation of the modified cooperative navigation scenario. From (**left**) to (**right**): attention entropy of Agent 1, Agent 2, and Agent 3.

To understand how the use of the attention mechanism contributes to the fault tolerance ability, we examined the “entropy” of the four attention weights from the attention heads in Figure 6. The initial value 0.69 represents the maximum possible entropy (i.e., uniform attention across all agents). Lower entropy indicates that the head is focusing on specific agents. We found that the agents are more willing to utilize head1 and head2, and each agent appears to use a different combination of the four heads.

**Figure 7:**
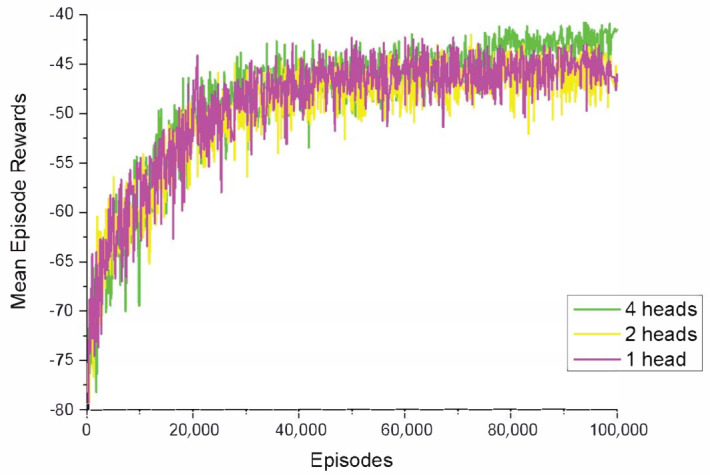
Learning curves of FT-Attn in the dynamic version of the modified cooperative navigation scenario with different numbers of attention heads.

To further test how the number of attention heads impacts the performance of FT-Attn, we recorded the learning curves for FT-Attn with different numbers of attention heads in the dynamic variant of the modified cooperative navigation scenario. From Figure 7, we observe that choosing 4 attention heads achieved the highest rewards, especially in the last training stage. Fewer attention heads did not achieve a satisfying performance, which was attributed to the abundant and comprehensive information focused on by the 4 attention heads. In concrete, the attention heads may divide their attention on the velocity, relative positions towards the landmarks, relative positions towards the other agents, and so on. We additionally tested the performance when the number of attention heads was set to 8. However, 8 attention heads did not affect the performance significantly, having no obvious difference from that of 2-head version. We determined the reason to be that the experience in the replay buffer suited the computational cost of 4 attention heads the most. To make the figure clear, we do not report the result of the 8-attention-head version here.

**Figure 8:**
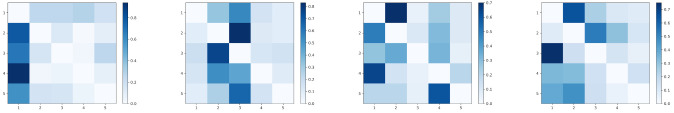
Attention weights generated by FT-Attn in the fixed case of the modified cooperative navigation scenario when *N* is set to 5. Scenario 1 to Scenario 4 are listed from **left** to **right**. Scenario 1: only the observation of Agent 1 is correct; Scenario 2: the observations of Agent 2, and Agent 3 are correct; Scenario 3: the observations of Agent 1, Agent 2, and Agent 4 are correct; Scenario 4: the observations of Agent 1, Agent 2, Agent 3, and Agent 4 are correct.

We further visualize the attention weights generated by FT-Attn to understand the interactions in the N = 5,L = 5 scenarios of the modified cooperative navigation environment containing a different number of gifted agents. Each agent’s attention weight was calculated from the heads that the agent appeared to use the most. We picked the scenarios containing four different combinations of the gifted agents (with the number of gifted agents set to 1, 2, 3, and 4, respectively) and show the related heat maps of the interaction matrix generated by FT-Attn in Figure 8. We can see that the agents acquired the ability to select the correct observations (self-attention is avoided in FT-Attn) since each agent’s attention weights on the gifted agents were larger than those on the faulted ones. Furthermore, the agents can also select the relevant and useful information among the correct observations. In other words, the agents did not pay uniform attention to the gifted agents, but instead with a fine discrimination, which demonstrated the superiority compared with the simple information-sharing mechanism in the MADDPG-M.

## 6. Discussion

The biggest limitation hindering our approach from being applied in practice is the number of agents supported. Our approach can currently support up to 16 agents due to the exponential explosion problem of the state space. We will exploit the parameter-sharing mechanism to learn efficient and effective communication for large-scale multi-agent cooperation. Besides, we will create more complicated environments where each agent should interact with a large group of agents where selective attention is needed. The setting naturally models real-life scenarios in which multiple agents are organized into clusters, such as a school, work, or family, where the agent needs to interact with a small number of agents from many groups.

To apply our approach in practice, a more useful representation is needed to avoid simply sharing the high-dimensional observations, which may contain redundant information. In practice, the environment is usually bandwidth-limited or there is a high communication cost. Therefore, the agents need to learn how to schedule themselves to suit the practical scenarios. In our future work, we will increase the number of agents and optimize our model to suit for limited-bandwidth scenarios. We believe that in such complicated scenarios, our approach will achieve a satisfactory performance combined with some advantages exhibited by other approaches.

## 7. Conclusions

We proposed a model, FT-Attn, for coping with the fault tolerance problem in multi-agent reinforcement learning systems. The key idea was to utilize the multihead attention mechanism to select the correct and useful information for estimating the critics. We evaluated the performance of FT-Attn in the modified cooperative navigation and modified predator and prey environments compared with the MADDPG-M, the previous state-of-the-art model dealing with extremely noisy environments. The empirical results showed that FT-Attn beat the baseline methods in some extremely noisy environments for both cooperative and competitive scenarios. Furthermore, FT-Attn does not rely on the prior knowledge about the noise intensity of the environment. In other words, FT-Attn can be directly utilized to learn in various kinds of noisy environments with no need to tune the configuration of the model. FT-Attn can effectively deal with the complex situation where an agent needs to reach multiple agents’ correct observation at the same time. We believe that adding our idea of fault tolerance will make the existing models much more valuable and practical. In our future work, we will make our approach more practical and further highlight the advantages of the fault tolerance ability in MARL systems.

## Figures and Tables

**Table 1:** Mean (standard deviations) episode rewards for all baselines in all 3 scenarios among 1000 episode tests. The larger the better, and the closer to the upper-bound the better. The bold best performance in each scenario does not consider the DDPG-OC since it reveals the upper-bound performance.

Method	Fixed	Alternating	Dynamic
Meta-agent	−39.95 ± 4.50	−51.42 ± 7.70	−60.98 ± 8.82
MADDPG	−54.00 ± 7.43	−58.67 ± 8.90	−63.44 ± 9.88
DDPG	−56.00 ± 8.96	−56.50 ± 8.51	−60.66 ± 8.68
MADDPG-M	**−39.73 ± 5.09**	−43.34 ± 7.29	−43.91 ± 7.75
**FT-Attn**	−40.89 ± 5.42	**−43.30 ± 7.83**	**−42.48 ± 7.36**
DDPG-OC (upper-bound)	−39.26 ± 4.45	−43.44± 5.92	−41.25 ± 5.24

**Table 2:** Mean (standard deviation) communication accuracies for FT-Attn in the three modified cooperative navigation scenarios when N = 3 among 1000 episode tests.

Method	Fixed	Alternating	Dynamic
MADDPG-M	**99.98% ± 0.02**	99.54% ± 0.56	88.82% ± 5.91
**FT-Attn**	99.23% ± 0.33	**99.68% ± 0.42**	**91.76% ± 5.34**

**Table 3:** Mean (standard deviations) episode rewards for all baselines in 4 scenarios among 1000 episode tests where the number of faulted agents is different in the N = 5 version of the modified cooperative navigation scenario. The larger the better, and the closer to the upper-bound the better. The bold best performance in each scenario does not consider the DDPG-OC since it reveals the upper-bound performance.

Method	1 Faulted	2 Faulted	3 Faulted	4 Faulted
Meta-agent	−66.31 ± 5.48	−66.98 ± 6.12	−67.92 ± 7.27	−69.73 ± 6.25
MADDPG	−72.50 ± 8.35	−72.83 ± 7.42	−72.79 ± 9.56	−72.68 ± 8.65
DDPG	−72.20 ± 7.12	−72.67 ± 5.79	−73.03 ± 5.62	−72.95 ± 5.43
MADDPG-M	−66.73± 5.43	−66.89 ± 8.43	−67.13 ± 6.64	**−66.95 ± 6.65**
**FT-Attn**	**−65.20 ± 4.38**	**−66.25 ± 8.62**	**−66.68 ± 6.57**	−67.00 ± 6.25
DDPG-OC (upper-bound)	−64.92 ± 4.23	−65.89 ± 7.73	−66.14 ± 6.42	−66.42 ± 7.78

## Data Availability

Not applicable.
